# Feasibility and Effectiveness of a Workplace-Adapted Mindfulness-Based Programme to Reduce Stress in Workers at a Private Sector Logistics Company: An Exploratory Mixed Methods Study

**DOI:** 10.3390/ijerph17051643

**Published:** 2020-03-03

**Authors:** Jesus Montero-Marin, Willem Kuyken, Virginia Gasión, Alberto Barceló-Soler, Lynda Rojas, Ana Manrique, Rosa Esteban, Javier García Campayo

**Affiliations:** 1 Department of Psychiatry, University of Oxford, Warneford Hospital, Oxford OX3 7JX, UK; jesus.monteromarin@psych.ox.ac.uk (J.M.-M.); willem.kuyken@psych.ox.ac.uk (W.K.); 2 Primary Care Prevention and Health Promotion Research Network (RedIAPP), 50009 Zaragoza, Spain; jgarcamp@gmail.com; 3 Institute for Health Research (IIS Aragón), 50009 Zaragoza, Spain; 4 Mindfulness Consultant, Parenthesis Consultants, 050031 Medellin, Colombia; lyndarojas@gmail.com; 5 Human Resources Department, Sese Group, 50014 Zaragoza, Spain; ana.manrique@gruposese.com (A.M.); rosa.esteban@gruposese.com (R.E.); 6 Miguel Servet University Hospital, University of Zaragoza, 50009 Zaragoza, Spain

**Keywords:** mindfulness, WA-MBP-LS, feasibility, stress, logistics sector, workplace

## Abstract

There is a high prevalence of stress in the logistics sector owing to very demanding, fast-paced and unpredictable tasks. Mindfulness-based programmes may reduce stress but require considerable practice. Our aim was to evaluate the feasibility and effectiveness of a shortened, workplace-adapted mindfulness-based programme for the logistics sector (WA-MBP-LS) for the purpose of reducing stress. A nonblinded, nonrandomised, two-arm controlled trial was conducted. The WA-MBP-LS (*n* = 32) consisted of six weekly 90-min mindfulness sessions. The control group (*n* = 36) attended a psycho-educational seminar. The Perceived Stress Scale (PSS) and Five Facets of Mindfulness Questionnaire (FFMQ) were measured at pretest, posttest and 6-month follow-up. Differences between groups were evaluated using mixed-effects models. Qualitative methods were used to analyse implementation issues. A 64.2% reduction was observed between initial volunteers and actual participants. Attrition at six-month follow-up was 45.6%. Participants attended a median of five sessions. Decreases in PSS favoured the WA-MBP-LS group at posttest and follow-up. FFMQ played a mediating role in PSS reductions. Barriers were disinterest, lack of programming, work overload and absences from work. Facilitators were curiosity, timing, company facilities and audio recordings. The WA-MBP-LS was feasible and effective in reducing stress, but more efforts to improve the practicalities of implementation are desirable.

## 1. Introduction

The intensification of the conditions imposed in the context of globalization by the dominant economic paradigm, including processes such as the rationalization and control of production and distribution of goods and services by the implementation of technological and bureaucratic systems, has resulted in important transformations in the structure of modern workplaces [1]. The logistics sector has acquired a prominent role in the context of the current economic paradigm. Whatever their nature, companies in this sector pursue two essential objectives: optimization of the constant flow of materials through agent networks, and the coordination of the necessary resources to achieve the effective delivery of products to customers.

The employees of logistics sector companies suffer from highly competitive, demanding, fast-paced, unpredictable and high-turnover job positions [2,3,4]. These employees are also under tight deadlines and pressure to prevent the late delivery of goods, and they are dependent on different factors, some of which are beyond their control, e.g., the weather, which can result in direct negative consequences such as the loss of earnings, sales and customer loyalty [5,6]. All of these conditions, in addition to the increasingly prevalent imposition of new forms of employment contracts, job insecurity, work intensification and poor work-life balance, require workers to make great psychological efforts in order to adapt to the changing demands of their jobs [7], and entail an enormous challenge for this type of worker and their ability to overcome such circumstances, which contributes to enhancing workers’ vulnerability to stress [8]. Work-related stress is the response people may have when presented with work demands and pressures that are not matched to their knowledge and skills, and which threaten their ability to cope [9]. It involves a psychosocial risk of major challenges to occupational health and safety [7]. However, research into the stress experienced by employees of private sector logistics companies is rather scarce at present [10].

Stress constitutes a considerable personal and economic burden for individuals, organizations and society at large. For individuals, stress increases the risk of suffering from mental health problems [11,12], health-impairing behaviours such as smoking or alcohol consumption [13,14], obesity [15], sleep disturbances [16], fatigue [17], musculoskeletal pain [18] and cardiovascular disease [19]. In organizational terms, stress has been related to reduced performance and productivity [20], poor safety outcomes [21], staff turnover [22], long-term sickness absences [23] and early retirement [24]. The cost of work-related stress to society is considerable [25], estimated at approximately €9.2 billion in the EU [26].

Mindfulness-based programmes (MBPs), which constitute a new approach within psychological medicine and are integrated into third-wave psychotherapies [27], have been expanded in recent years and have proven effective for a variety of health-related outcomes and populations [28,29,30]. The first MBP, mindfulness-based stress reduction (MBSR), was originally developed to reduce stress in clinical settings [31], although a number of reviews and meta-analyses on the salutary effects of MBPs have also demonstrated positive outcomes in nonclinical fields, including the workplace [32,33,34,35,36,37,38,39]. In fact, it has been recently concluded that stress reduction is a promising target area of MBPs for promoting workplace wellness [40], and some studies have also shown improvements in other outcomes such as emotion regulation, job satisfaction, sleep quality, heart rate variability, mental well-being and performance [41,42,43,44]. Nevertheless, the majority of studies in this field have been conducted using samples of public sector employees, principally health care professions and teaching staff, while very few have been conducted in the private sector, where there may be greater difficulties associated with the implementation of these types of programmes owing to particular practice requirements [38].

MBPs are a type of mind-body intervention that aim to train the mind to adopt nonjudgemental, present-focused awareness [45]. This type of training for the self-regulation of attention might decrease stress and facilitate well-being by helping the practitioner to learn to stay in the present moment with curiosity and acceptance [31,46]. By training the mind to realise how intention and behaviour are formed in the stream of sensory-perceptual events, negative thoughts, sensations, emotions and behaviours begin to be seen as something changeable, allowing practitioners to experience the world in a less threatening way [44,46]. Two different mechanisms through which mindfulness could promote and facilitate psychological adjustment have been proposed [47]: mindfulness might directly facilitate clarity to current experience, promoting an accurate point of view of reality without the discriminatory and prejudiced thought filter [48]; and it might indirectly enhance self-regulated functioning from attentional sensitivity to somatic, psychological and environmental cues [49].

MBPs require participants to be quite disciplined in the practice of meditation exercises and to invest a substantial amount of time [50]. This characteristic of MBPs might represent a barrier in terms of suitability if we are to implement this type of intervention in the workplace, particularly in the context of the private sector, and even more so in the logistics sector, where work obligations can make participation and practice very difficult [37]. For this reason, original standard programmes, such as MBSR, which usually comprise 8 weekly 150-min sessions plus a one-day retreat, as well as 20–45 min of individual practice per day [51], need to be reduced when applied in the workplace, giving rise to shortened versions of MBP [41,42,43,52]. It has been observed that a reduced MBP consisting of only half of the standard version could obtain similar effect sizes to those of the standard MBP in terms of trait mindfulness, affectivity, anxiety and depressive symptomatology, compared to waiting list controls when applied to nonclinical populations such as undergraduate students [53]. In view of this, further research studies are warranted to address specific application contexts (e.g., workplace occupational domains such as those of for-profit logistics companies in the private sector), in which brief adaptions of standard MBPs have not previously been tested, in order to make them more accessible to larger numbers of participants without losing their effectiveness [36].

Against this background, the main objective of this study was twofold: to evaluate (a) the feasibility and (b) potential effectiveness of a workplace-adapted MBP for reducing stress in workers of a private sector logistics company. The workplace-adapted MBP for the logistics sector (WA-MBP-LS) was developed by the authors to be a brief, suitable, cost-effective, evidence-based and replicable MBP curriculum to enhance mindfulness and reduce stress among employees of for-profit, privately-owned logistics operators. The WA-MBP-LS was inspired by the theory and practice of contemplative traditions and psychological science; it seeks to address and relieve human distress and promote well-being by training individuals to engage in a new way of relating to experiences based on a present moment orientation, proposes to develop greater attentional self-regulation and values such as compassion, and engages participants in mindfulness meditation practice. Therefore, the WA-MBP-LS is an MBP curriculum that meets the most recently proposed definition for an MBP [54]. It was skilfully adapted to the workplace so that it can be delivered onsite at logistics platforms among company employees. Exploring the possibilities of applying an MBP to reduce stress in new populations and specific workplace contexts such as those of private sector logistics companies, is relevant for advancing our understanding of the scope and possible limitations of these types of mind-body programmes in settings that are in need of intervention; however, difficulties might also be encountered in terms of the implementation process, owing to job-related idiosyncratic aspects, e.g., time pressure constraints [55].

Specifically, with regard to the employees of a for-profit, privately-owned logistics company, we aimed to explore: (1) the feasibility of the recruitment procedure and retention of the initial group of volunteers until completion of the WA-MBP-LS programme; (2) the acceptability of the WA-MBP-LS by means of the rates of adherence to the practice; (3) the data collection procedure in terms of the response rate to surveys; (4) the potential effectiveness of the WA-MBP-LS at reducing perceived stress, as well as improving mental well-being, job satisfaction and trait mindfulness; (5) the extent to which participation in the WA-MBP-LS intervention influences gains in trait mindfulness, and whether these gains in the condition of mindfulness are associated with improvements in perceived stress, mental well-being and job satisfaction; and (6) the barriers and facilitators that might determine the successful implementation of the WA-MBP-LS intervention in the logistics sector, using qualitative methods.

## 2. Materials and Methods

### 2.1. Design

As a mixed-methods, naturalistic feasibility trial with a nonblinded, nonrandomised, controlled, two-arm and parallel-group design, the WA-MBP-LS was offered to a group whose levels of perceived stress, well-being, job satisfaction and trait mindfulness were measured at baseline, postintervention and six-month follow-up, and compared to control participants who only attended a psycho-educational seminar. Both groups were interviewed about aspects of the programme implementation process. The study was revised and approved by the corresponding company’s human resources section (01/06/2018). This study was conducted in accordance with the Declaration of Helsinki of 1975, revised in 2013. The data (totally anonymised) used to produce the study results and descriptions of the project are available in full on the following open access repository (more information for replication studies are available upon request): https://www.openicpsr.org/openicpsr/project/108743/version/V8/view.

### 2.2. Study Sample and Procedure

The study was incorporated into a voluntary training course to reduce perceived stress that was offered by the human resources department of a privately-owned Spanish logistics company to the 300 people working at their head office in February 2018. The course was free of charge for employees and consisted of six weekly 90-min mindfulness-based sessions integrated into their work schedule, with the possibility of 15 min of daily practice also taking place within working hours. Participants received no reimbursement for their participation in the study. The concept of mindfulness, as well as its possible applications to an occupational context in the logistics sector was explained in two 30-min, face-to-face recruitment group sessions held in two shifts at the company’s facilities and facilitated by the company’s human resources manager and a psychologist representing the research group. Attendees at these informative sessions were able to ask questions to clarify any doubts after having been given the opportunity to review information sheets detailing the possible benefits of the programme, the confidentiality with which the data would be processed and the ethical implications of participation, and which contained a link to the online survey. A total of 190 employees agreed to participate in the study on the condition they would be able to choose the specific group in which they would participate. Randomization was therefore not viable. The experimental group consisted of the WA-MBP-LS (its contents are explained in detail below), and the control condition was based only on a psycho-educational seminar with a duration of 2 h on the transactional model of stress and the salutary effects of MBPs regarding coping with stressful situations in the workplace.

Since this was an exploratory pilot study, a strict sample size calculation was not performed. However, we aimed for approximately 50 participants (25 in each group) because this would be a large enough sample to inform us about the practical aspects under study and to reach statistical significance for perceived stress using a 95% confidence interval and a statistical power of 80% in a two-tailed contrast with a large effect size (d = 0.80), as had previously been obtained using mind-body stress reduction programmes in the workplace [44]. The inclusion criteria were as follows: a) ability to understand spoken/written Spanish; b) online submission of a signed written informed consent form; and c) completion of the online baseline evaluation before beginning the intervention. Of all the volunteers who initially agreed to participate, a total of 122 workers did not complete the baseline assessment and were therefore excluded from the study. The participants were allowed to choose the group in which they wished to participate, with 32 of them included in the WA-MBP-LS condition and 36 in the control group, for a total of 68 participants at baseline.

#### Workplace-Adapted Mindfulness-Based Programme for the Logistics Sector (WA-MBP-LS)

The WA-MBP-LS is a stress management intervention based upon the principles and practices of mindfulness for organizations [56], but specifically customised to ‘just-in-time’ contexts of application in which seconds count and where stock breaks in assembly lines represent economic penalties and losses in terms of the reputation of the company. These organizational contexts are dynamic environments that require individuals to make a series of interdependent decisions in real time, to show flexibility in the face of changes, creativity in the resolution of contingencies, and a resilient attitude in order to maintain efficiency by providing immediate solutions despite difficulties. This is a type of setting in which developing nonjudgemental observation might have a practical bearing because high levels of attention are needed to solve the challenges that continuously arise. The practice of mindfulness gradually allows individuals to realise that events can be more fluid. In other words, even stressful situations at work that generate apparently negative behaviours, thoughts and sensations come to be seen as more flexible and changeable, allowing them to shift their experience by learning to pay attention to the present moment, with a curious and accepting attitude, and higher concentration over long periods of time [46,57]. While these processes are not necessarily conscious ones, their results could allow individuals to experience the workplace in a less stressful way.

The WA-MBP-LS condition was divided into two subgroups, each with 16 participants, and sessions were held in the workplace in March–April and May–June 2018 by a trained mindfulness teacher, certified in MBSR and with 5 years’ experience. The teacher in charge of delivering the intervention also contributed to the development of the specific adaptations to the WA-MBP-LS, and was also the person who presented the psycho-educational seminar attended by the control group. The entire programme was delivered at the employee workplaces. The WA-MBP-LS is an adaptation of the MBSR programme [31]. The original standard programme was reduced to six weekly 90-min sessions. No full-day retreat was offered, but it included the possibility of 15-min, audio-recorded daily practices. This reduction in terms of the length of sessions and daily practices, as well as the absence of a day of silence retreat (full day), meant a shortening by approximately half of the amount of practice of the original programme. This level of intensity is within the range of practice that has shown effective results on well-being outcomes among nonclinical samples in previous research [53], and it was considered an acceptable level of practice that might be included in the schedule of privately-owned companies in the logistics sector, characterised by important time pressure constraints.

The WA-MBP-LS sessions were eminently practical and interactive, combining explanations about the transactional stress model, as well as processes of the mind and the body, with attention practices, conscious movements and meditation exercises. The exercises were relatively brief (5–15 min) and were specifically designed to be used in workplace settings. The dynamic of the practices included alternation between moments of silence and periods of collective exploration that involved inquiring strategies for facing stress and difficult situations, and discussion on how to practically apply these strategies in the professional sphere related to the logistics sector. At the same time, daily practices were also held in the workplace. For these daily practice sessions, the company allotted two 15-min timeframes in the morning, from 8:00 to 8:15 a.m. and from 8:15 to 8:30 a.m. The daily practice sessions were conducted using audio-recorded exercises that had been previously explained and put into practice under supervision during the training sessions. Once the programme was concluded, the participants were encouraged to continue with the practice at home on an individual basis, but no more interventions involving the MBP teacher or any electronic device or Internet platform were made available after that time in order to evaluate the impact of the WA-MBP-LS as an exploratory, low-intensity starting point for implementation. Some details of the WA-MBP-LS sessions, contents and daily practice exercises can be found in Table 1.

### 2.3. Measures

#### 2.3.1. Socio-Demographic and Occupational Characteristics

An online data collection procedure was used. The survey included socio-demographic variables at baseline, such as age, sex, relationship (yes, no), number of children, residence (urban, rural), level of education (primary, secondary, university), weekly working hours, years of service in the company, sick leave in the last year (yes, no), type of contract (temporary, permanent), income satisfaction (not satisfied, slightly satisfied, moderately satisfied, very satisfied), minutes of vigorous weekly physical activity, and mindfulness practice during the previous six months (yes, no).

#### 2.3.2. Attrition and Acceptability

Recruitment of and attrition from the total group of workers through the initial group of interested volunteers until the final completion group were assessed by recording the flow of participants throughout the study. Acceptability was measured by recording the number of programme sessions attended and attendance at daily practices. Data collection was evaluated by means of the number of online records completed at pretest, posttest and six-month follow-up.

#### 2.3.3. Perceived Stress

Stress levels were measured at pretest, posttest and six-month follow-up by the ‘Perceived Stress Scale’ (PSS) [58]. The PSS is a self-report instrument that evaluates the level of perceived stress during the previous month. This scale consists of 10 items asking about the frequency of thoughts and feelings experienced using a Likert-type scale with 5 response options, between 0 (‘never’) and 4 (‘very often’). The total scale is obtained as the sum of all the items and ranges between 0 and 40; higher scores correspond to higher levels of perceived stress. The PSS Spanish version has demonstrated good psychometrics in previous research [59]. Cronbach’s alpha in the present study was α = 0.90.

#### 2.3.4. Mental Well-Being

The short ‘Warwick-Edinburgh Mental Wellbeing Scale’ (SWEMWB) [60] was used to assess mental well-being at pretest, posttest and six-month follow-up. It is a self-report questionnaire consisting of 7 items that are ranked by a Likert-type scale from 1 (‘never’) to 5 (‘always’). This scale considers mental health from a positive perspective, more in relation to functioning than to feelings; as such, it covers eudaimonic aspects of psychological functioning—i.e., well-being as something related to the development of a person’s abilities, challenges, purpose in life and growth—but it also includes some hedonic aspects, i.e., related to the presence of positive affectivity and satisfaction with life. The total scale is the sum of all the items and presents a range from 7 to 35, where higher scores correspond to higher levels of well-being. This scale has demonstrated appropriate psychometrics in its Spanish version [61]. Cronbach’s alpha in the present study was α = 0.80.

#### 2.3.5. Job Satisfaction

Job satisfaction was evaluated using the ‘Job Satisfaction Scale’ (JSS) at pretest, posttest and six-month follow-up. The JSS is a self-report questionnaire that was designed for exploring general occupational aspects of job satisfaction in the present study. It asks questions on five topics: relationship with superiors, relationships with other workers, level of assigned responsibility, acknowledgement obtained for work well done, and attention paid by the company to worker suggestions. The degree of satisfaction for each of these items is assessed using a Likert-type scale with 7 response options, from 1 (‘very unsatisfied’) to 7 (‘very satisfied’). A total job satisfaction score is calculated as the sum of all the items, ranging between 5 and 35 points; higher scores correspond to higher levels of job satisfaction. The factorial validity of the JSS in the present study presented very good fit to the data (CFI = 0.998; TLI = 0.999; RMSEA = 0.064; SRMR = 0.015), also with an adequate internal consistency of α = 0.88.

#### 2.3.6. Mindfulness

Mindfulness as a trait was assessed by the ‘Five Facet Mindfulness Questionnaire’ (FFMQ) [62] at pretest, posttest and six-month follow-up. The FFMQ is a 39-item, self-report measure of trait mindfulness that is based on the following five skills: observing, describing, acting with awareness, and nonjudging of and nonreactivity to inner experience. Observing refers to the ability to realise external and internal experiences (e.g., sensations, thoughts and emotions); describing is the ability to label internal experiences with words; acting with awareness includes focusing intently on the activities of the present moment; nonjudging refers to the adoption of a nonevaluative posture towards the feelings and thoughts that are experienced; and nonreactivity involves relating to internal experiences by allowing feelings and thoughts to reach and leave the focus of consciousness without being trapped by them. Respondents indicate on a 5-point Likert-type scale the degree to which each item is true for them, from 1 (‘never true’) to 5 (‘always true’). Higher scores indicate higher levels of trait mindfulness. A total FFMQ score is obtained as a result of the sum of all the items, ranging between 39 and 195. The FFMQ Spanish version has shown appropriate psychometric properties [63]; in the present study, Cronbach’s alpha internal consistence value for the total scale was α = 0.92.

### 2.4. Statistical Analysis

First, socio-demographic data were described at baseline by frequencies (%), medians (inter-quartile range, IQR) or means (standard deviation, SD), and the study groups were compared by means of a chi-square test (or Fisher’s test when necessary), Mann-Whitney test or *t*-test, respectively, depending on the level of measurement and distribution of each socio-demographic variable.

Recruitment and attrition were described using frequencies (%). The number of WA-MBP-LS sessions and daily practice sessions attended were reflected using frequencies (%), medians (IQR) and means (SD). As a guideline, we expected a rate of attrition of approximately 50% between the initial group of volunteers to the final group of participants at baseline, and of approximately 30% from the final group of participants at baseline to the final group at the six-month follow-up [64]. In terms of acceptability, a mean value of half of the sessions attended (≈3.0) was considered appropriate but in need of improvement, and a mean value of 2/3 of the sessions attended (≈4.0) was considered feasible [65]. Given the high time pressure suffered by workers in the logistics sector, a mean value of 1 daily practice session at workplace facilities per week during programme implementation was defined as appropriate but in need of improvement, and a mean value of ≥ 2 daily practices per week during this period was defined as feasible. Values lower than those specified were considered nonfeasible results.

The effectiveness of the WA-MBP-LS in reducing perceived stress (PSS) and improving well-being (SWEMWBS), job satisfaction (JSS) and trait mindfulness (FFMQ) compared to that for the control group was analysed using the variables in a continuous manner. We developed a repeated measures design with an intention to treat approach using mixed-effects regression models, including time as an independent variable and subjects as a random effect. The restricted maximum likelihood method was used, which produces unbiased estimates of parameters when using small sample sizes or unbalanced data [66]. Nonstandardised slopes, i.e., 95% confidence intervals (95% CIs), were calculated by adjusting the baseline scores and those socio-demographic variables with significant differences at pretest. The ‘group x time’ interaction was considered to determine whether possible differences between groups remained consistent over time. Within-group tests were performed for each group separately in order to evaluate their specific changes over time. Effect sizes (ESs) were assessed using Cohen’s d statistic, calculated by the combined SD weighing the mean difference [67]. Cohen [68] defined ESs as small when d ≤ 0.2, as medium when d = 0.5, and as large when d ≥ 0.8. Sensitivity analyses were conducted to assess the effects of missing data and to evaluate how robust our effectiveness analyses were at follow-up. Missing values were replaced using multiple imputations based on chained equations after ensuring that the data were missing at random. We developed an exploratory comparison of pre–post improvements between subgroups of participants in the WA-MBP-LS condition in terms of sex and level of education following the same analytical procedure, and also explored whether age and weekly working hours were related to pre–post change scores using Pearson’s r coefficients.

We evaluated the clinical relevance of improvements in PSS by differentiating participants into ‘responders’ vs ‘nonresponders’ to the intervention. To do so, we used the baseline 25th percentile from our total sample as a cut-off criterion to classify participants [58,59], determining that they had reached low perceived stress (i.e., responders) if they scored under the specified cut-off at posttest and six-month follow-up. This classification was used to calculate the absolute risk reduction and the number needed to treat (NNT) in the WA-MBP-LS condition compared to the control group. NNT refers to the number of participants who need to be treated with the intervention (i.e., rather than the control condition) for one additional participant to benefit; a 95% CI was calculated for each NNT.

We evaluated the extent to which the level of participation in the programme, i.e., the total hours of participation in WA-MBP-LS sessions and daily practice sessions from pretest to posttest treatment, was related to pre–post and pre–follow-up gains in trait mindfulness (FFMQ), perceived stress (PSS), mental well-being (SWEMWBS) and job satisfaction (JSS) using Pearson’s r coefficients. In addition, we also used Pearson’s r correlations to evaluate to what extent pre–post gains in FFMQ were associated with pre–follow-up improvements in PSS, SWEMWBS and JSS.

The sample size of the present study was not primarily estimated to assess mediating effects. Thus, the potential mediating role of FFMQ on PSS, SWEMWBS and JSS was evaluated by merging subjects inside a within-participant path-analytical framework in order to gain statistical power [69,70]. For this purpose, we explored the relationships between the repeated-measures factor (independent variable), the FFMQ pre–post differential scores (mediating variable), and the PSS, SWEMWBS and JSS pre–follow-up differential scores (dependent variables) using ordinary least squares (OLS) analysis with unstandardised path estimates from linear regression coefficients. It was estimated that about 68 participants would allow us to identify significant indirect effects (IEs) as the product of paths ‘a’ and ‘b’—which has been shown to approximate the power of bootstrap estimates [71]—with a statistical power of 0.80, assuming the existence of intermediate effects with a standardised value of about 0.35 in both ‘a’ and ‘b’ paths of the mediating model, and of 0.45 in the ‘c’ path (direct effects controlling for the IEs), and therefore considering a partial mediation scenario with room for other possible mediators. The unstandardised regression coefficients and their corresponding standard error for IEs were calculated, as were their 95% bias corrected CIs based on 10,000 bootstrap samples. This test is applied to overcome possible problems of asymmetry in the distribution of the IEs [72]. IEs are considered significant when their 95% CI does not include zero. Multiple determination coefficients (R^2^) were used to estimate the ES of the mediating models, with values of 0.00 = null effect, 0.14 = small effect, 0.39 = medium effect, and 0.59 = large effect [73].

No interim analyses were performed prior to finishing the complete study trial. An alpha level of 0.05 was set using a two-tailed test. Analyses were carried out using the STATA v12.0 and the IBM SPSS v19.0 statistical software packages.

### 2.5. Qualitative Analysis

To understand the challenges associated with implementing the WA-MBP-LS intervention in a private sector logistics company, a psychologist trained in qualitative methods held in-depth interviews with participants after follow-up in order to assess: (a) why people chose to participate in the experimental group vs the control group; (b) why they continued attending or stopped attending sessions; and (c) why their attendance was regular or sporadic. Interviews were conducted until data saturation and were recorded and transcribed verbatim. The textual corpus was extracted for analysis using qualitative methods. A content analysis was performed iteratively by two independent researchers to identify the emerging categories from which all the transcripts could be coded [74]. As a first step, a general thematic framework analysis identified the main topics. Secondly, we determined which aspects of the general framework might act as barriers to and facilitators for implementation of the WA-MBP-LS intervention in the logistics context. Finally, we described each of the emerging categories by using empirical segments of the verbatim recordings and transcriptions, establishing possible relationships between them to discover the underlying core and secondary dimensions, which were subsequently defined. The necessary ability of the category system to adequately capture all of textual materials, as well as whether each category was comprehensive and exclusive of the others [75], was subject to debate and solved by consensus (in case of discrepancies, a third researcher was consulted). The data analysis was performed with the Maxqda 2018 software package.

## 3. Results

### 3.1. Participant Flow and Compliance

**Figure 1:**
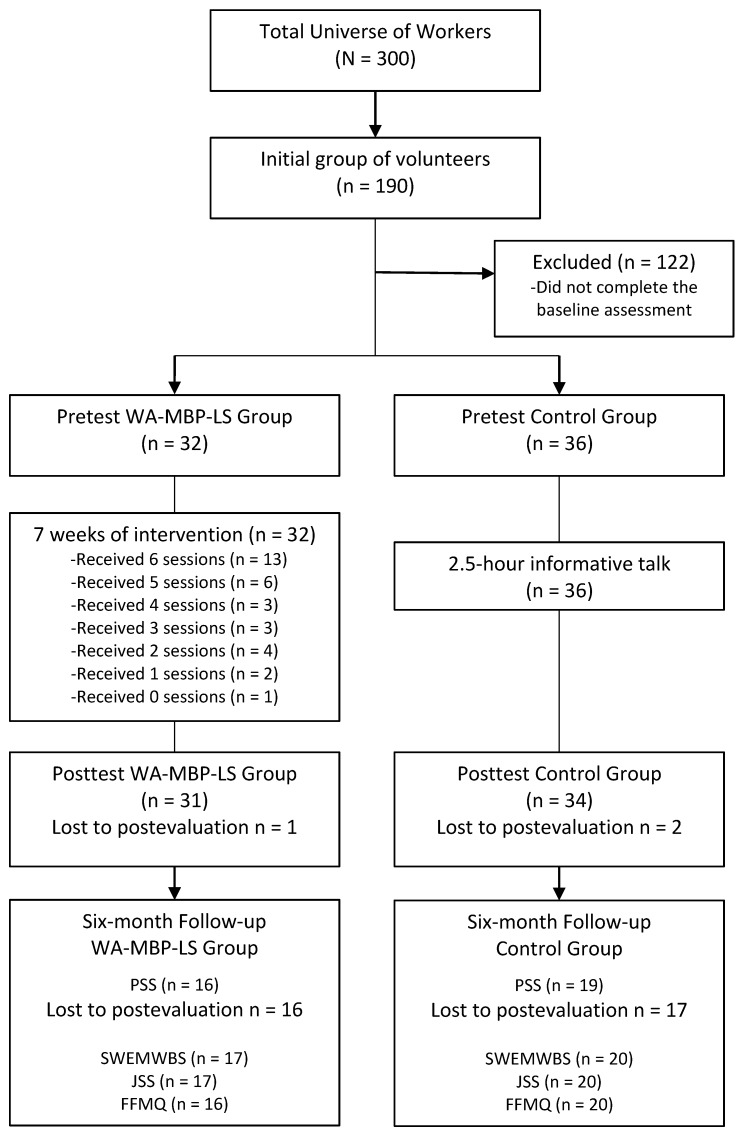
Flow of study participants.

The CONSORT recommendations for reporting pilot and feasibility trials were followed [76]. Figure 1 reflects the flow of participants through the study. Out of a total of 300 employees of the logistics company, 110 declined to participate. Of the 190 remaining subjects who were potential volunteers, 122 were excluded because they did not complete the baseline evaluation. Thus, 68 subjects were finally included in the study (35.8% of the initial volunteers), 32 of whom went on to the WA-MBP-LS arm and 36 of whom formed the control group attending a psycho-educational seminar. The 32 subjects participating in the WA-MBP-LS arm attended a mean of 4.34 (SD = 1.86) training sessions (median = 5; Q_1_–Q_3_ = 3–6). A total of 13 participants (40.6%) attended all the training sessions, while 9 (28.2%) attended 4 or 5 sessions, and 10 participants (31.3%) attended < 4 sessions (1 participant did not attend any training sessions). The total daily practices held presented a mean of 8.44 (SD = 7.78) practice sessions (median = 6; Q_1_–Q_3_ = 2–15). A total of 12 participants (37.5%) attended ≥ 10 daily practice sessions, while 7 participants (21.9%) attended ≥ 4 but <10 sessions, and 13 participants (40.6%) attended < 4 sessions (4 participants did not attend any daily practice sessions). The mean of total hours participating in both training sessions and daily practice sessions from pretreatment to posttreatment in the WA-MBP-LS group was 8.63 h (SD = 4.12), with a minimum of 0.50 h and a maximum of 15.25 h. All the control subjects attended the two-hour psycho-educational seminar.

The PSS questionnaire was completed by 31 subjects in the WA-MBP-LS group and by 34 subjects in the control group at postintervention, and it was completed by 16 subjects in the WA-MBP-LS group and 19 subjects in the control group at six-month follow-up, representing 95.6% and 51.5% of the participants who entered the study, respectively (see Figure 1 for the other outcomes). At the six-month follow-up, 6 (31.6%) participants in the control group and 11 participants (64.7%) in the WA-MBP-LS condition had been practising mindfulness after posttreatment (χ^2^ = 4.46; *p* = 0.035). No baseline-level differences in the socio-demographic data or in the psychological outcomes were observed either at pretest or posttest between those who completed the survey at the six-month follow-up and those who did not. Thus, dropouts were considered to be random [77].

### 3.2. Group Baseline Characteristics

Slightly more than half of the participants were women in their late thirties, with partners, no children, and a university education, living in an urban setting (Table 2). The mean working week was roughly 40 h. The participants had accumulated approximately 6 years of service in the company, and the majority had not been on sick leave in the previous year. The majority of the participants’ contracts were permanent, and more than half of the participants were moderately satisfied with their income. The mean weekly time spent on vigorous physical activity was less than an hour, and only a minority had practised meditation during the previous six months. There were significant between-group differences at baseline in terms of age (*p* = 0.025) and meditation practice during the previous six months (*p* = 0.026); thus, both of these variables were controlled in the effectiveness analyses.

The mean PSS score for the total group at baseline was 18.28 (SD = 7.00), ranging between 1 and 31 (median = 19; IQR = 14–23). Therefore, we considered a score of ≤13 as the cut-off point to classify participants as ‘responders’ vs ‘nonresponders’ to the intervention in terms of perceived stress. This same cut-off point had already been used in previous studies to differentiate participants with low levels of perceived stress using the PSS questionnaire [78,79,80]. There were no significant differences between the WA-MBP-LS and control groups in the number of subjects below this classification criterion at baseline [WA-MBP-LS: 5 (15.6%); controls: 11 (30.6%); χ^2^ = 2.10; *p* = 0.147].

### 3.3. Effectiveness of the WA-MBP-LS Intervention

**Figure 2:**
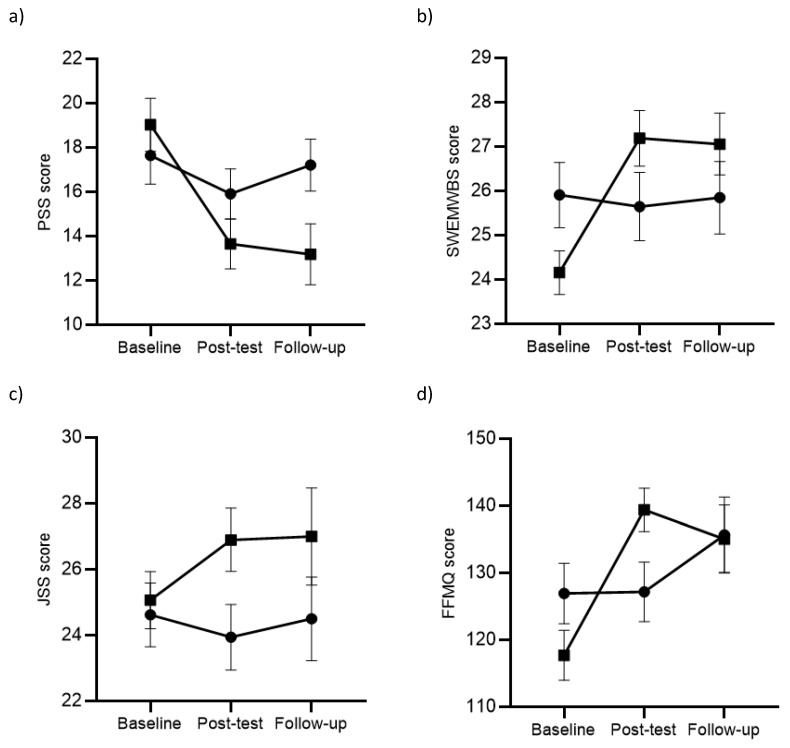
Graphical representation of WA-MBP-LS and control groups on outcomes over time. *Note*: WA-MBP-LS: Workplace-adapted mindfulness-based programme for the logistics sector. (**a**) PSS: Perceived Stress Scale. (**b**) SWEMWBS: Warwick-Edinburgh Mental Wellbeing Scale. (**c**) JSS: Job Satisfaction Scale. (**d**) FFMQ: Five Facet Mindfulness Questionnaire. ■ WA-MBP-LS group. ● Control group.

Table 3 shows the descriptive and between-group analyses for PSS, SWEMWBS, JSS and FFMQ (Figure 2 presents a graphical representation of outcomes over time). As can be seen, there were significant between-group differences in PSS, with the WA-MBP-LS participants performing better than controls at posttest and six-month follow-up, with moderate and high ESs, respectively. Within-group tests showed that participants in the WA-MBP-LS group significantly improved on PSS at posttest (B = −5.39, 95% CI = −7.04–−3.74, d = −0.80; z = −6.39, *p* < 0.001), and follow-up (B = −5.82, 95% CI = −7.82–−3.82, d = −1.02; z = −5.69, *p* < 0.001). On the contrary, participants in the control group did not show significant within-group improvements at posttest, although there was a trend (B = −1.74, 95% CI = −3.50–−0.03, d = −0.60; z = −1.93, *p* = 0.054) and follow-up (B = 0.31, 95% CI = −1.78 − 2.40, d = −0.12; z = 0.29, *p* = 0.770).

There were also significant between-group differences in the SWEMWBS, with the WA-MBP-LS participants performing better than controls at posttest and at six-month follow-up, with high ESs (Table 3). Within-group tests showed that participants in the WA-MBP-LS group significantly improved on SWEMWBS at posttest (B = 3.03, 95% CI = 2.07–4.00, d = 1.10, z = 6.16, *p* < 0.001) and follow-up (B = 2.51, 95% CI = 1.35–3.68, d = 1.30, z = 4.24, *p* < 0.001). Participants in the control group did not show significant within-group improvements at posttest (B = −0.27, 95% CI = −1.49–0.96, d = −0.07, z = −0.42, *p* = 0.671) and follow-up (B = −0.14, 95% CI = −1.56–1.29, d = −0.02, z = −0.19, *p* = 0.852).

We observed a trend with a moderate ES at posttest on the JSS, favouring the WA-MBP-LS participants, and differences were significant at six-month follow-up (Table 3). Within-group tests showed that participants in the WA-MBP-LS group significantly improved on JSS at posttest (B = 1.84, 95% CI = 0.19–3.49, d = 0.42, z = 2.19, *p* = 0.029), and they showed a trend very close to significance and of similar ES at follow-up (B = 1.94, 95% CI = −0.03–3.90, d = 0.48, z = 1.94, *p* = 0.053). Participants in the control group did not show significant changes at posttest (B = −0.68, 95% CI = −2.59–1.23, d = −0.11, z = −0.69, *p* = 0.487) and follow-up (B = −0.97, 95% CI = −3.20–1.26, d = −0.03, z = −0.85, *p* = 0.393).

Finally, we found significant differences in the FFMQ total score, favouring the WA-MBP-LS participants at posttest and at six-month follow-up, with high and moderately low ESs, respectively (Table 3). Within-group tests showed that participants in the WA-MBP-LS group significantly improved on FFMQ at posttest (B = 21.71, 95% CI = 15.34–28.08, d = 1.07, z = 6.68, *p* < 0.001) and follow-up (B = 18.02, 95% CI = 10.10–25.93, d = 0.89, z = 4.46, *p* < 0.001). Participants in the control arm did not show significant improvements at posttest (B = 0.24, 95% CI = −4.33–4.80, d = 0.02, z = 0.10, *p* = 0.920), but they did at follow-up (B = 6.88, 95% CI = 1.57–12.19, d = 0.58, z = 2.54, *p* = 0.011).

Models with imputed missing values at the six-month follow-up showed similar results for the regression coefficients, reinforcing the significant differences observed in PSS, SWEMWBS and JSS scores, but not in the FFMQ total scores, which presented a considerable reduction in the regression coefficient and showed a nonsignificant difference between groups (Table 3).

Considering only the WA-MBP-LS group, we observed that men, compared to women, obtained greater pre–post reductions of moderate ES in PSS, and although they were not significant, they did show a trend (B = −2.73, 95% CI = −5.51–0.05, d = −0.42; z = −1.92, *p* = 0.054). No other noticeable differences in outcomes were observed according to sex or level of education in the WA-MBP-LS group. On the other hand, we found that older participants showed greater pre–post reductions in PSS, with no significant but moderate effects that showed a trend (r = 0.34; *p* = 0.063). Finally, we observed that longer working hours per week were significantly related to less pre–post SWEMWBS improvement, with moderate effects (r = −0.36; *p* = 0.045). No other noticeable relationships were observed regarding the other outcomes.

### 3.4. Absolute Risk Reduction and Number Needed to Treat

We calculated the absolute risk reduction and NNT to determine the clinical significance of improvements in perceived stress (PSS) of the WA-MBP-LS group compared to controls. A total of 54.8% of the WA-MBP-LS participants and 38.2% of the controls [17 of 31 (WA-MBP-LS) and 13 of 34 (controls)] reached the criterion of ≤13 on the PSS after treatment. Therefore, the absolute risk reduction in the WA-MBP-LS condition increased by 16.6% (95% CI includes zero) compared to that in the control group, with an NNT = 7 (95% CI includes zero). On the other hand, 68.8% of the WA-MBP-LS participants and 26.3% of the controls [11 of 16 (WA-MBP-LS) and 5 of 19 (controls)] who completed the follow-up assessment reached the criterion of ≤ 13 on the PSS at follow-up. Therefore, the absolute risk reduction in the WA-MBP-LS increased by 42.4% (95% CI = 13.3–72.6) compared to that in the control group, with an NNT = 3 (95% CI = 1.4–8.1).

### 3.5. Level of Practice and Gains in Mindfulness, Stress, Well-Being and Job Satisfaction

The total time (training sessions and daily practice sessions) invested in participating in the programme was significantly related to pre–post gains in FFMQ (r = 0.59, *p* < 0.001), PSS (r = −0.27, *p* = 0.031) and SWEMWBS (r = 0.41, *p* = 0.001), but it was not significantly related to JSS (r = 0.13, *p* = 0.320). On the other hand, the time invested in participating in the programme was also significantly related to pre–follow-up gains in FFMQ (r = 0.49, *p* = 0.002), PSS (r = −0.45, *p* = 0.007) and SWEMWBS (r = 0.41, *p* = 0.012), but it was not significantly related to JSS, although there was a trend (r = 0.30, *p* = 0.075).

### 3.6. Mediating Role of Mindfulness on Perceived Stress, Well-Being and Job Satisfaction

Pre–post change scores in FFMQ were significantly related to pre–follow-up change scores in PSS (r = 0.37, *p* = 0.029) and SWEMWBS (r = 0.33, *p* = 0.048), but they were not significantly related to change scores on the JSS (r = 0.26, *p* = 0.116). As can be seen in Table 4, pre–post increases in FFMQ (a = 11.91, *p* = 0.003) played a mediating role for pre–follow-up improvements in PSS (b = −0.10, *p* = 0.031; IE = −1.20, 95% CI = −2.57, −0.20) and SWEMWBS (b = 0.04, *p* = 0.048; IE = 0.50, 95% CI = 0.05, 1.13) with small to medium effects. The percentage of the effect of the study condition mediated through the FFMQ was 49.4% on PSS, and of 47.6% on SWEMWBS. Pre–post increases in FFMQ did not significantly mediate pre–follow-up improvements on the JSS (b = 0.06, *p* = 0.122; IE = 0.64, 95% CI = −0.14, 1.48).

### 3.7. Barriers and Facilitators to Implementation of the WA-MBP-LS Programme

**Figure 3:**
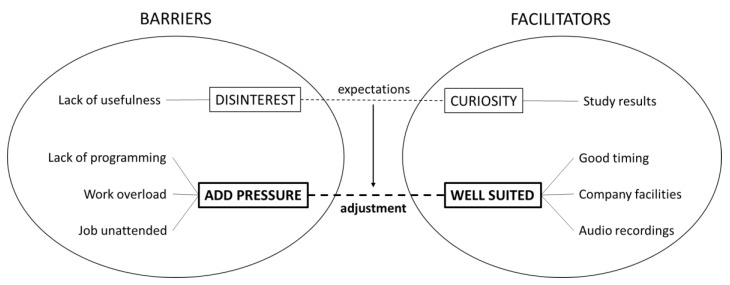
Dimensions, categories and properties determining the implementation of the WA-MBP-LS. *Note*: WA-MBP-LS: Workplace-adapted mindfulness-based programme for the logistics sector.

A total of 12 workers were interviewed in depth until data saturation (*n* = 6 WA-MBP-LS participants and *n* = 6 controls), with a total mean age of 39.58 years (SD = 9.02), 50% of whom were women, with a mean for years of service with the company of 7.17 (SD = 5.80). Figure 3 provides a graphic representation of the structure of relationships found between the core and secondary emergent dimensions, as well as its constituent categories and properties determining the implementation of WA-MBP-LS according to employees’ perspectives, while Table 5 contains transcribed quotes regarding the results of all the properties found.

The qualitative analysis of the verbatim recording and transcription showed that ‘lack of programming’ when adjusting the course schedule, peaks of ‘work overload’ and the need to leave the ‘job unattended’ with no replacement personnel were responsible for ‘adding pressure’ to the workers, and thus, functioned as important barriers. Controls specifically said that the first hour of the morning, i.e., the time in which sessions were held, was a critical moment in terms of time pressure and workload, and that they did not want to leave the job at that time. On the other hand, ‘good timing’ within working hours, as well as using ‘company facilities’ and the ‘availability of audio recordings’ to practise individually at peak work times and at home were seen as a well-suited configuration that facilitated the implementation of the programme. The two poles of ‘add pressure’ and ‘well suited’ were the two extremes of the underlying ‘adjustment’ dimension, which was the core category from which the whole speech was structured, pointing to the importance of considering specific job-related aspects, such as the moment of delivery, possible workload peaks, the need for staff reinforcement, being able to choose between different practice times (e.g., morning, afternoon), using company facilities inside and outside of working hours, and the availability of audio materials to allow for more flexible individual practice.

Parallel to this, there was another dimension that crossed between the barrier and facilitator zones. This dimension reflected individual ‘expectations’ at the time of choosing participation, and they could be negative, e.g., ‘disinterest’, with ideas of ‘lack of usefulness’ of mindfulness interventions, probably due to ignorance of the field. However, they also could be positive, e.g., ‘curiosity’, with interest in knowing what mindfulness is and to what extent it could be of help for them according to ‘study results’. WA-MBP-LS participants decided to choose to be active participants as a result of a certain attitude of curiosity that was aroused in them. Interestingly for this group, being overloaded was precisely the incentive to try to overcome a stressful job, but one important difference with the other group was that they were able to manage in order to allow the programme to suit their job positions well. Thus, expectations reflect a secondary dimension of adjustment that should be considered in the first phases of implementation (e.g., informative talks), with a possible influence on the adjustment of the WA-MBP-LS programme, which, in turn, is a core dimension that requires specific work from the human resources department. Table 6 shows the theoretical definitions for these two dimensions of the qualitative model.

## 4. Discussion

The purpose of this study was to evaluate the feasibility of delivering the WA-MBP-LS and its potential effectiveness in reducing perceived stress in employees of a for-profit, privately-owned logistics company, i.e., a context in which high levels of stress are generally experienced with high impacts on health [8,10]. Most of the experimental evidence on mindfulness programmes has been generated within the context of basic research with nonworkplace samples or with public sector employees, such as healthcare providers and teachers, raising generalizability questions and doubts that need to be addressed [36,37,38]. To overcome this, we adapted a mindfulness-based programme to the specified target population and context of application, i.e., for employees of a private sector logistics company, with the intention of evaluating the practicalities that can facilitate the refinement of various aspects of MBP implementation, and also to assess the preliminary effectiveness of such a reduced MBP through direct experience, before moving to a more comprehensive, fully powered investigation.

We first observed that within the imposed study conditions, the employee volunteers were not willing to be randomised between groups; they preferred to choose to join a group instead of being randomly assigned to the intervention or the control groups, and thus, their expectations may have determined the study findings in part. This aspect does not emerge explicitly in noncontrolled trials, but it needs to be considered in terms of whether more robust studies should be conducted [81,82]. In fact, the results of our qualitative analyses suggested negative expectations in the form of disinterest and, more particularly, through ideas of lack of usefulness of mindfulness programmes, which may have been an important barrier that negatively influenced the implementation processes. Other important reasons for participants’ unwillingness to be randomised were because of added pressure to the worker on the job, for instance by the lack of a possibility to temporarily choose or change the time of the course in relation to peaks of greater workload, and also of having to leave the job uncovered by another person. In short, the possibility of adequately managing one’s workload during the particular intervention programme was an aspect that could cause differences in the willingness to participate actively, i.e., precisely those workers who were most in need of the intervention could have more difficulties in accessing the programme; this should be given special consideration [83]. Interestingly, the control group was composed of employee volunteers who were significantly younger, and therefore, holding a less secure position in the structure of the company, and had previously had more practical mindfulness experience, and thus, perhaps had ‘nothing new to learn’ from the programme.

Initial interest in participation suggested that it might be very feasible to deliver the WA-MBP-LS to employee volunteers in the workplace context under study. However, only about 36% of potential volunteers completed the baseline assessment, which signifies a limited reach. Reasons for not completing the baseline assessment and not being involved with the programme could be related to the aforementioned barriers of a perceived lack of usefulness and added pressure. Although the response rate to questionnaires was very high at posttreatment, it was approximately 55% at the six-month follow-up, which was less than expected for MBPs in other settings [30,64], suggesting possible extra difficulties in this specific context of application. One possible strategy to increase interest and compliance, thereby reducing attrition, might be to offer an individualised personal feedback report to participants after completing the study, in addition to the anonymised group report that is usually presented to employers [81]. Moreover, the use of face-to-face measurements by employing an independent assessor to collect self-report data instead of the online procedure may also be more productive in this regard. These procedures might increase the importance and centrality of the research for participants, thereby facilitating recruitment and retention. Furthermore, the use of stepped-wedge designs, in which clusters of subjects are randomly selected to receive the intervention after a period in which no subjects are exposed to the programme, could be especially adaptable to the needs of the workplace of a for-profit, privately-owned company in the logistics sector, facilitating recruitment and the collection of more complete and richer data, and more particularly, reconciling the need to attend mindfulness sessions and evaluations with idiosyncratic workplace constraints [84,85,86,87].

We observed that the mean number of training sessions and daily practice sessions attended were 4.3 and 8.4, respectively, which could be considered appropriate acceptability values, although there might be some room for improvement [64]. Improvements in acceptability could be achieved, for instance, by implementing a virtual community of support through a WhatsApp group administered by the WA-MBP-LS teacher, who could be entrusted with the task of offering daily reminders to complete the practice along with motivating messages [88]. In general, MBPs suffer from high rates of attrition [30,64], and failure to consolidate practice may undermine the benefits related to the intervention [89,90]. It has been observed that an individual’s exposure to the programme might facilitate acceptability [91]. This suggests that introducing a small practice session during the presentation of the programme might improve expectations, facilitating curiosity and also initial interest, and in turn, recruitment. This could also be important in order to improve follow-up rates, which might be possible by increasing acceptability through adherence to practice. According to our results, a well-suited programme might be achieved by facilitators such as good timing of the programme, the use of company facilities both inside and outside of working hours, even with different shifts, and the promotion of a variety of materials, e.g., audio recordings for individual use.

We found that employee volunteers who participated in the WA-MBP-LS experienced moderate reductions in perceived stress after receiving the intervention, compared to controls who attended a brief psycho-educational seminar, with six-month sustainability and large effects at follow-up. We also observed that the WA-MBP-LS might have a potential clinical relevance to reduce perceived stress at the six-month follow-up in the WA-MBP-LS intervention group when compared to controls. These preliminary findings are in line with previous research on the general impact of MBPs among treated participants in comparison with waiting list controls [30], and they are also similar to other workplace MBPs regarding reductions obtained in perceived stress [38]. In addition, we also observed that the WA-MBP-LS might contribute to improving other outcomes, such as general mental well-being, with large ESs, and job satisfaction, with moderate ESs. Considering the level of practice reached by participants, this supports the idea of a ‘parsimonious intervention’, in the sense that it may be beneficial at a minimum dose for different psychological outcomes, improving distinct aspects of the general psychological functioning of workers [36]. In addition, the level of participation in the programme was significantly and linearly related to gains in mindfulness, perceived stress and overall mental well-being. Gains in mindfulness seemed to be a mechanism of change for improvements in perceived stress and overall mental well-being. This reinforces the theoretical assumptions that explain the efficacy of this type of training to enhance psychological adjustment by means of self-regulation of attention and acceptance processes [31,44,46]. Nevertheless, the influence of the level of participation and the possible mediating role of mindfulness on job satisfaction issues was not entirely clear, and further research is needed using more powerful designs to detect possible lower effects.

MBPs are increasingly being implemented in work settings [40]. However, they are usually truncated versions of standardised programmes that are adapted to the specific features of each context of application, which is why research on optimal designs are important in the search for maximum efficacy and sustainability [41,42,43]. In terms of mindfulness as a trait, we observed large improvements in the WA-MBP-LS participants compared to controls at posttest but moderately low effects at the six-month follow-up. The latter result was due to unexpected improvements in trait mindfulness in the control group at follow-up, and we believe these improvements might be a consequence of the informative talk they received in the seminar and of the external practice of mindfulness exercises by some of these control subjects outside of the workplace programme between posttest and follow-up. It seems some interest in mindfulness practices could be activated as a consequence of the talk the control group received, and possibly by sharing experiences with the WA-MBP-LS participants. Here, again, the use of stepped-wedge designs, in which all the subjects are eventually treated, could help to understand this result and manage this situation, alleviating possible ethical concerns derived from not being completely treated, and further favouring greater sustainability of the programme [84,85,86]. In general, we observed that the minimum dose of treatment required to obtain positive changes in mindfulness as a trait seemed to be achieved during the intervention. Nevertheless, we also observed possible moderating effects of sex, age and weekly working hours. Although underpowered to analyse this, our results suggest that women and younger participants could be benefiting less from the WA-MBP-LS in terms of stress reduction. The possible causes of this, e.g., structural issues, require further research. We have also observed that those participants with longer working hours could be improving well-being to a lesser extent than those with shorter hours. This might indicate that positive outcomes are harder to improve on under this condition, but it needs to be confirmed and explained with more detail in future research. Only very recently have studies examined the implementation of MBIs in health [92] and education [93]. These studies have used an established theoretical framework to map the key contextual and facilitating factors that support implementation, with some overlap (e.g., the importance of “champions”) and some differences (e.g., importance of clinical guidelines in health and “perceptions of mindfulness” in education). Future research could use our results and that explanatory framework to better understand how to effectively implement an MBP in workplace contexts.

### Limitations and Strengths

As we have stated previously, further research is needed involving more powerful and higher-quality designs in order to provide a more precise idea of the possibilities of the WA-MBP-LS and its potential role in extending the observed improvements to other organizational and work life areas. Firstly, participants who did not complete the baseline assessment might differ from those who did, and this could involve generalizability issues. Moreover, even considering the impossibility of enrol participants based on randomization principles, this remains as a limitation of the present study. Other limitations of this study include the use of a small set of self-report questionnaires, with special emphasis on overall measures of perceived stress and mental well-being, and not accounting for other additional and more specific work-related variables such as job performance, organizational climate and culture, burnout, absenteeism and presenteeism. This limitation must be given specific consideration in future research to offer a deeper understanding of the possible impact of the WA-MBP-LS on workers in for-profit privately owned companies in the logistics sector [81]. In general, addressing more complex designs, ensuring intervention integrity [90], and promoting a more flexible training through other modes of delivery, including mobile applications or online platforms that could reinforce individual levels of practice, would be desirable [88,94]. Finally, a cost-effectiveness analysis of the WA-MBP-LS would provide an additional economic perspective on feasibility. The main strengths of the study were the collection of long-term follow-up data, which is important in order to evaluate the sustainability of effects and possible mechanisms of change, as well as the integration of the programme within working hours, facilitating participation. Another strength was the use of qualitative methods to understand the implementation processes from the perspectives of the actual participants.

## 5. Conclusions

In general, our results suggest that the WA-MBP-LS could be feasible when applied to for-profit, private sector logistics companies, but that it should be amended to optimise the procedure in order to achieve greater rates of recruitment and retention. The WA-MBP-LS seemed to be acceptable in this professional context, although there may be room for improving compliance. The application of WA-MBP-LS was related to reductions in perceived stress and improvements in mental well-being and job satisfaction, but it is not yet clear to what extent this programme could improve other work-specific outcomes, and further studies using randomised controlled trials are needed to investigate whether changes found can be directly attributed to the WA-MBP-LS. All in all, our data reflect the procedure of only one pilot study, but the findings may serve as a template for more complex and powerful future designs to test other intervention formats, intensities, outcomes, etc., considering the necessary adaptations to suit different professions in order to gain flexibility when applying the programme.

## Figures and Tables

**Table 1:** Workplace-adapted mindfulness-based programme for the logistics sector (WA-MBP-LS).

Sessions	Contents	Daily Practices
1. Mindfulness vs automatic pilot	Presentation of teachers/participants Group norms, expectations What is mindfulness? Raisin exercise Body scan exercise Three-minute practice Work-related stress in the logistics sector	Body scan Three-minute practice
2. Living in the present and not in the mind	Body scan exercise Review of current practice and practices at home Analysis of difficulties What to do with the body and the mind Meditation in breathing: (a) three anchor points; (b) nuclear practice; (c) breath counting Formal practice at work in the logistics sector	Body scan Mindful breathing
3. Increasing our attention	Informal practices Review of current practices and of the week Seeing what is seen and hearing what is heard Deal with thoughts Exercise: ’hello’, ‘thank you’ and ‘goodbye’ Informal practice at work in the logistics sector	Mindful breathing Mindfulness of a daily activity
4. Understanding how mindfulness works	Seated meditation (breathing, body, sounds, thoughts and consciousness without choice) Review of current and home practice How does mindfulness work? Mindfulness in movement and walking meditation Three-minute period with thoughts Mindful attitudes at work in the logistics sector	Seated meditation Three-minute period Mindful walking and mindfulness in movement
5. Values approach	Breathing exercise Review of current practice and of the week How do you feel before the end of the course? 3 stages: challenge, disappointment and acceptance. How does it work? (in pairs) Concept of values in life and work-life Resilience and energy balance at work in the logistics sector	Mindful breathing or body scan Regular three-minute periods in adverse situations Walking meditation
6. Compassion: caring for me and others	Breathing exercise Review of current practice and of the week What is compassion? Self-support patterns in difficult situations Compassionate gestures, phrases and confrontations Self-care at work in the logistics sector Revision of the course. How to keep up practice	Mindful breathing or body scan Compassionate coping Three-minute practice

**Table 2:** Socio-demographic and labour data of participants at baseline.

Variables	Total (*n* = 68)	Control (*n* = 36)	WA-MBP-LS (*n* = 32)	*p*
Age, (range: 25–60 years), *Mean* (SD)	38.56 (7.64)	36.61 (7.76)	40.75 (6.99)	0.025
Sex, women, n *(%)*	44 (64.7)	25 (69.4)	19 (59.4)	0.386
Relationship, yes, *n (%)*	44 (64.7)	24 (66.7)	20 (62.5)	0.720
Children, *median* (Q_1_-Q_3_)	0 (0-2)	0 (0-2)	1 (0–2)	0.421
Residence, urban, *n (%)*	62 (91.2)	33 (91.7)	29 (90.6)	0.999
Education level, *n (%)*				
Primary	4 (5.9)	3 (8.3)	1 (3.1)	0.462
Secondary	18 (26.5)	11 (30.6)	7 (21.9)	
University	46 (67.6)	22 (61.1)	24 (75.0)	
Weekly working hours, *mean* (SD)	42.13 (4.24)	41.64 (4.04)	42.69 (4.46)	0.313
Years of service, *mean* (SD)	6.51 (5.11)	5.66 (5.21)	7.33 (4.97)	0.212
Sick leave last year, yes, *n (%)*	11 (16.2)	6 (16.7)	5 (15.6)	0.907
Contract type, temporary, *n (%)*	8 (11.8)	6 (16.7)	2 (6.3)	0.266
Income satisfaction, *n (%)*				
Not satisfied	1 (1.5)	0 (0.0)	1 (3.1)	0.108
Slightly satisfied	22 (32.4)	9 (25.0)	13 (40.6)	
Moderately satisfied	42 (61.8)	24 (66.7)	18 (56.3)	
Very satisfied	3 (4.4)	3 (8.3)	0 (0.0)	
Minutes of weekly vigorous physical activity, *mean* (SD)	49.33 (70.88)	52.00 (78.39)	46.41 (62.76)	0.750
Meditation practice last 6 months, yes, *n (%)*	6 (8.8)	6 (16.7)	0 (0)	0.026

Note: WA-MBP-LS: Workplace-adapted mindfulness-based programme for the logistics sector. Data are presented as the means (SD), medians (Q1–Q3) or frequencies (%), according to each variable. p: value of the statistical significance associated with the contrast between groups, using a *t*-test, Mann-Whitney test, or χ^2^ (or Fisher) test.

**Table 3:** Between-group analyses of perceived stress, mental well-being, job satisfaction and mindfulness.

	WA−MBP−LS			Control			Between−Group Analyses		
	n	Mn (SD)		n	Mn (SD)		*d*	*Z* (*p*)	*B* (95% CI)
**PSS**									
Pre−test	31	19.03 (6.63)		34	17.65 (7.58)				
Post−test	31	13.65 (6.28)		34	15.91 (6.62)		−0.52	−2.93 (0.003)	−3.65 (−6.10–−1.21)
Follow−up	16	13.19 (5.65)		19	17.21 (5.25)		−0.75	−4.15 (<0.001)	−6.20 (−9.13–−3.28)
								*−5.08 (<0.001)*	*−4.03 (−5.58–−2.47)*
**SWEMWBS**									
Pre−test	31	24.16 (2.73)		34	25.91 (4.31)				
Post−test	31	27.19 (3.48)		34	25.65 (4.49)		0.91	4.05 (<0.001)	3.30 (1.70–4.89)
Follow−up	17	27.06 (2.88)		20	25.85 (3.65)		0.82	2.79 (0.005)	2.67 (0.80–4.55)
								*7.79 (<0.001)*	*2.92 (2.19–3.66)*
**JSS**									
Pre−test	31	25.07 (4.81)		34	24.62 (5.66)				
Post−test	31	26.90 (5.31)		34	23.94 (5.80)		0.48	1.92 (0.052)	2.52 (−0.05–5.08)
Follow−up	17	27.00 (6.07)		20	24.50 (5.68)		0.40	2.00 (0.045)	3.07 (0.05–6.09)
								*3.81 (<0.001)*	*2.40 (1.16–3.63)*
**FFMQ**									
Pre−test	31	117.71 (20.80)		31	126.94 (26.33)				
Post−test	31	139.42 (18.05)		31	127.18 (25.84)		0.90	5.44 (<0.001)	21.47 (13.74–29.21)
Follow−up	16	135.06 (21.00)		20	135.70 (25.19)		0.36	2.33 (0.020)	11.12 (1.76–20.47)
								*−0.17 (0.864)*	*−0.33 (−4.12–3.45)*

Note: Models developed by repeated measures (RM) linear mixed-effects regression analysis controlling for the baseline, age and meditation practice in the previous six months. SWEMWBS: Short Warwick-Edinburgh Mental Wellbeing Scale. PSS: Perceived Stress Scale. JSS: Job Satisfaction Scale. FFMQ: Five Facet Mindfulness Scale. *Italic*: sensitivity analyses by imputing missing values at six-month follow-up using chained equations. WA-MBP-LS: Workplace-adapted mindfulness-based programme for the logistics sector group. Control: Control group.

**Table 4:** Mediation analyses of mindfulness on perceived stress, mental wellbeing and job satisfaction.

		*Direct Effects*						*Indirect Effects*			
Outcome	*R* ^2^	*Path*	*B*	*SE*	*t*	*p*	*95% CI*	*Path*	*Boot.*	*SE*	*95% CI*
PSS (*n* = 35)	0.17	a b c’	11.91 −0.10 −1.23	3.67 0.04 1.09	3.25 −2.26 −1.14	0.003 0.031 0.265	4.46, 19.37 −0.19, −0.01 −3.45, 0.98	ab	−1.20	0.61	−2.57, −0.20
SWEMWBS (*n* = 37)	0.18	a b c’	11.46 0.04 0.55	3.48 0.02 0.51	3.29 2.05 1.08	0.002 0.048 0.290	4.40, 18.52 0.01, 0.09 −0.49, 1.59	ab	0.50	0.28	0.05, 1.13
JSS (*n* = 37)	0.07	a b c’	11.46 0.06 −0.37	3.48 0.04 0.83	3.29 1.58 −0.44	0.002 0.122 0.664	4.40, 18.52 −0.02, 0.13 −2.06, 1.33	ab	0.64	0.41	−0.14, 1.48

Note: PSS: Perceived Stress Scale. SWEMWBS: Warwick-Edinburgh Mental Wellbeing Scale. JSS: Job Satisfaction Scale.

**Table 5:** Quotes regarding the WA-MBP-LS implementation from participants point of view.

Dimensions (Centrality)	Categories (Role)	Properties	Quotes
Adjustment (core)	Add pressure (barrier)	Lack of programming	Q3: ‘In my department, the hours of the day where you have dead times are not exactly first thing in the morning’ (Control, 50 years old, man, 15 years of service with the company).
		Work overload	Q2: ‘I have had a lot of work, and that’s why I wasn’t able to reconcile the two things, I was in a moment of a lot of work and that’s why I decided not to do the course’ (Control, 28 years old, woman, 4 years of service with the company).
		Job unattended	Q3: ’At that time there was no one to replace me, I could have taken part but I felt bad leaving work’ (Control, 43 years old, woman, 4 years of service with the company).
	Well suited (facilitator)	Good timing	Q4: ‘I decided to participate in the intervention because the schedule suited me very well’ (WA-MBP-LS group, 36 years old, man, 6 years of service with the company).
		Company facilities	Q5: ‘The fact that it was held at the company’s facilities and during working hours was very valuable’ (WA-MBP-LS group, 38 years old, woman, 3 years of service with the company).
		Audio recordings	Q6: ‘The audio recordings made it easy for me to practise, sometimes I even did exercises at night’ (WA-MBP-LS group, 36 years old, man, 7 years of service with the company).
Expectations (secondary)	Disinterest (barrier)	Lack of usefulness	Q7: ‘I don’t believe in these things, and that’s why I didn’t take part. I don’t think it offers any benefits’ (Control, 42 years old, man, 13 years of service with the company).
	Curiosity (facilitator)	Study results	Q8: ‘I was curious to know what mindfulness was, and the fact that a study was done was especially interesting because it can provide real data for the company’ (WA-MBP-LS group, 48 years old, woman, 7 years of service with the company).

Note: WA-MBP-LS: Workplace-adapted mindfulness-based programme for the logistics sector.

**Table 6:** Theoretical definitions for the dimensions of the qualitative model.

Dimensions	Importance	Definitions
Adjustment	Core	Specific job-related aspects of the WA-MBP-LS implementation, such as the moment of delivery, possible workload peaks, the need for reinforcement staff, being able to choose between different practice shifts, using company facilities inside and outside of working hours, and the availability of audio materials to allow a flexible and adaptable individual practice.
Expectations	Secondary	Sub-dimension of adjustment that reflects individual preferences regarding the WA-MBP-LS programme, from general disinterest to curiosity regarding mindfulness practices and study results.

Note: WA-MBP-LS: Workplace-adapted mindfulness-based programme for the logistics sector.
